# Analysis of within-individual variation in extrapair paternity in blue tits (*Cyanistes caeruleus*) shows low repeatability and little effect of changes in neighborhood

**DOI:** 10.1093/beheco/araa069

**Published:** 2020-10-01

**Authors:** Kristina B Beck, Mihai Valcu, Bart Kempenaers

**Affiliations:** Department of Behavioural Ecology and Evolutionary Genetics, Max Planck Institute for Ornithology, Eberhard Gwinner Str. 7, 82319 Seewiesen, Germany

**Keywords:** alternative mating strategies, extrapair paternity, mating system, neighborhood, repeatability, social environment

## Abstract

Many studies investigated variation in the frequency of extrapair paternity (EPP) among individuals. However, our understanding of within-individual variation in EPP remains limited. Here, we comprehensively investigate variation in EPP at the within-individual level in a population of blue tits (*Cyanistes caeruleus*). Our study is based on parentage data comprising >10 000 genotyped offspring across 11 breeding seasons. First, we examined the repeatability of the occurrence of EPP, the number of extrapair offspring, the number of extrapair partners, and the occurrence of paternity loss using data from males and females that bred in multiple years. Second, we tested whether within-individual changes in EPP between breeding seasons relate to between-year changes in the local social environment. Repeatabilities were generally low but significant for the occurrence and number of extrapair young in females and for whether a male sired extrapair young or not. We found no evidence that the presence of the former social partner or changes in the proportion of familiar individuals or in phenotypic traits of the neighbors influenced changes in levels of EPP in females. However, in adult males, a decrease in the average body size of male neighbors was associated with higher extrapair siring success. If confirmed, this result suggests that the competitive ability of a male relative to its neighbors influences his extrapair mating success. We suggest that alternative hypotheses, including the idea that within-individual changes in EPP are due to “chance events” rather than changes in an individual’s social breeding environment, deserve more consideration.

## Teaser

Why do individuals vary in extrapair behavior? We examined the within-individual variation in extrapair paternity (EPP) across 11 seasons in a population of blue tits. We show that measures of EPP show low repeatability and that changes in the breeding environment explained only little of the changes in EPP. Thus, alternative hypotheses, including the idea that variation in EPP depends on “chance events,” should be considered.

## INTRODUCTION

Animals often show within-population variation in mating behavior. This variation can be caused by several underlying mechanisms: from genetically determined strategies (e.g., Tsubaki 2003; Küpper et al. 2016) via age-dependent mating tactics (e.g., Richard et al. 2005; Apio et al. 2007) to individual flexibility in response to the (social) environment (e.g., Leary et al. 2008; Mulrey et al. 2015).

A well-studied example of such variation is the occurrence of extrapair paternity (EPP) in birds. Although the majority of species are socially monogamous, copulations outside the social pair bond are widespread and cause varying levels of EPP (Griffith et al. 2002; Westneat and Stewart 2003; Brouwer and Griffith 2019). Extrapair copulations will typically benefit males because they can sire additional offspring, but the adaptive value of extrapair behavior for females remains controversial (Forstmeier et al. 2014; Whittingham and Dunn 2016; Plaza et al. 2019). To understand the evolution of EPP and its consequences for sexual selection (Webster et al. 1995; Schlicht and Kempenaers 2013), we need to find out why males vary in extrapair siring success and why females vary in how many of their eggs are sired by their social mate.

In general, extrapair behavior and its outcome can be considered individual-specific traits. This would be the case 1) if males and females differ in their propensity to be promiscuous (e.g., if extrapair behavior is heritable; Reid et al. 2010; Forstmeier et al. 2011; Germain et al. 2018), 2) if some males are better at competing for extrapair copulations (e.g., because they are larger; Weatherhead and Boag 1995; Schlicht et al. 2015a) or at siring extrapair offspring (e.g., because they produce more or more competitive sperm; Moller and Briskie 1995; González-Solís and Becker 2002; Knief et al. 2017), or 3) if females consistently choose particular (high-quality or highly attractive) males for extrapair copulations (Hasselquist et al. 1996; Whittingham and Dunn 2016). Within-individual consistency in levels of EPP can also arise if 4) individuals consistently breed in an environmental context that increases opportunities for extrapair behavior (Schlicht et al. 2015a; Biagolini-Jr et al. 2017).

Within-individual consistency of EPP has been examined by considering multiple measures of the trait for a set of individuals (e.g., across several years) and calculating the repeatability of the trait, defined as the proportion of the total variance that is due to between-individual variation (Lessells and Boag 1987; Bell et al. 2009). The consistency of EPP traits can provide information about the potential strength of sexual selection and past studies often examined the repeatability of female extrapair behavior as an indirect estimate of heritability (Boake 1989). Studies on a variety of songbirds reported the repeatability in the number of extrapair young produced or sired (e.g., Dietrich et al. 2004: *R*_Females_ = 0.30, *R*_Males_ = 0.29; Reid et al. 2010: *R*_Females_ = 0.13; Whittingham et al. 2006: *R*_Females_ = 0.83), the number of extrapair sires (e.g., Whittingham et al. 2006: *R*_Females_ = 0.73), and the occurrence of EPP (Charmantier and Blondel 2003: no evidence for repeatability in females and males; Møller and Tegelström 1997: *R*_Females_ = 0.72). Although measures of EPP are repeatable to some extent, the estimates vary considerably and the underlying causes remain unclear.

Some studies report a low or modest repeatability of EPP, suggesting that much of the variation is due to changing circumstances that relate to opportunities to engage in extrapair copulations or to success in siring extrapair offspring. First, individual characteristics might change over time. For instance, many studies have shown that young (yearling) males have lower extrapair siring success compared to older (adult) males (Cleasby and Nakagawa 2012; Hsu et al. 2017; Michálková et al. 2019). Second, the environmental context relevant for extrapair behavior can change considerably for an individual between breeding attempts. For example, levels of EPP may vary with aspects of the current (social) environment, such as breeding synchrony (Stutchbury and Morton 1995; Saino et al. 1999; Thusius et al. 2001), breeding density (Westneat et al. 1990; Dunn et al. 1994; Araya-Ajoy et al. 2015), the density of the vegetation (Biagolini-Jr et al. 2017), the presence of predators (Santema et al. 2019), or whether an individual breeds with the same or a different social partner (within-pair repeatability; Dietrich et al., 2004). However, most studies that examined the effects of the local environment on EPP considered among-individual variation within a given breeding season (for our study population, see, e.g., Schlicht et al. 2015a; Beck et al. 2020) instead of within-individual variation across seasons.

Here, we comprehensively investigate within-individual variation in patterns of EPP across successive breeding attempts in a population of blue tits (*Cyanistes caeruleus*) comprising 11 breeding seasons. First, we examined to what extent the occurrence of EPP is a repeatable, individual-specific trait for males and females. Second, we investigated whether within-individual changes in measures of EPP between years can be explained by between-year changes in the local breeding environment of a focal individual. This approach allows disentangling effects of individual-specific, “intrinsic” traits from those due to the local breeding environment and may, thus, help to understand variation in EPP. For example, EPP levels may be highly repeatable because individuals breed consistently in an environment favoring extrapair copulations (i.e., a high repeatability in the breeding environment). In such cases, we expect that between-year changes in the local environment will explain the observed within-individual variation in EPP. If there is no effect of the local environment, it is more likely that the occurrence or frequency of EPP reflects one or more individual-specific “intrinsic” traits. Conversely, if levels of EPP show low repeatability and changes in the local environment explain the observed within-individual variation, EPP is a highly context-dependent trait.

We considered three relevant contexts in which the breeding environment of a focal individual can change between years, whereby we specifically focus on the social context: territory size, the identity of the social partner, and the local neighborhood (for an overview of all variables included for males and females, their interpretation, and our predictions, see Table 1 and Supplementary Figure S1). Extrapair behavior is inherently an interaction between multiple individuals (i.e., the male or female, its social partner, and the potential extrapair mates) but how the social environment affects patterns of EPP has rarely been examined (Petrie and Kempenaers 1998; Westneat and Stewart 2003; Maldonado-Chaparro et al. 2018).

**Table 1:** Overview of the variables reflecting the local environmental context in which EPP occurs and predictions about how they can explain between-season changes in the expression of EPP

Explanatory variable	Definition^a^	Background	Predictions	
			Female	Male
∆ Territory size	year_*x* + 1_/year_*x*_	Individuals possessing larger territories may be less likely to engage in extra-pair copulations (EPCs) because the larger distance might limit the encounter probability with potential extrapair mates (Westneat and Sherman 1997; Thusius et al. 2001; Westneat and Mays 2005; but see Schlicht et al. 2015a).	An increase in territory size is associated with less EPP	
∆ Number of neighbors	year_*x* + 1_/year_*x*_	A higher local breeding density (i.e., a higher number of neighbors) should increase opportunities for EPCs because more potential extrapair partners are in close proximity (Westneat and Sherman 1997; Thusius et al. 2001; Schlicht et al. 2015a).	An increase in the number of neighbors will lead to more EPP	
∆ Tarsus length of social partner	year_*x* + 1 −_ year_*x*_	Larger males are more likely to gain EPP (Akçay and Roughgarden 2007) and less likely to lose paternity (Kempenaers et al. 1992; but see Strohbach et al. 1998).	Females paired with a larger social partner in year_*x* + 1_ will have less EPP as larger males might be better at mate guarding or are of higher quality.	—
Consistency of social partner	Same or different social partner in year_*x* + 1_	Remaining with the same mate over multiple years can be seen as a sign of pair compatibility (Ihle et al. 2015), which might reduce extrapair behavior.	Individuals that keep the same social partner might have less EPP in year_*x* + 1_.	
∆ Familiar neighbors	year_*x* + 1 −_ year_*x*_	Familiarity among neighbors can facilitate extraterritorial visits through reduced territorial aggression (Beletsky and Orians 1989; Grabowska-Zhang et al. 2011) and familiar individuals (including former extrapair or social mate) might be more likely to visit each other.	Individuals with more familiar male or female neighbors will have more EPP in year_*x* + 1_.	
∆ Proportion of yearling male neighbors	year_x+1 −_ year_x_	Adult males are more likely to gain EPP (Akçay and Roughgarden 2007). More adult males in the neighborhood might reduce the chances for a male to gain EPP and increase the probability that the female has EPY.	If the proportion of yearling males increases, females will have less EPP.	If the proportion of yearling males increases, the focal males will have more EPP.
∆ Average tarsus length of male neighbors	year_x+1 −_ year_x_	Larger males are more likely to gain EPP (Akçay and Roughgarden 2007). Larger males in the neighborhood might reduce the chances for a male to gain EPP and increase the probability that the female has EPY.	If the average size of neighboring males increases, females will have more EPP.	If the average size of neighboring males decreases, males will have more EPP (less competitive environment).
∆ Proportion of yearling female neighbors	year_x+1 −_ year_x_	Adult females may be more aggressive toward intruding neighbor females than yearling females. More adult females in the neighborhood might reduce the chances for a female to obtain EPCs.	If the proportion of yearling females increases, females will have more EPP	—
∆ Average tarsus length of female neighbors	year_*x* + 1 −_ year_*x*_	Larger females may be more successful in displacing intruding neighbor females than smaller females. More large females in the neighborhood might reduce the chances for a female to obtain EPCs.	If the average size of neighboring females increases, females will have less EPP.	—
Previous social partner	Previous social partner present in neighborhood in year_*x* + 1_ or not	Blue tits engage in EPCs with previous social partners (Gilsenan et al. 2017).	Individuals that have a previous social partner in their close neighborhood might have more EPP.	
Previous extrapair partner	Previous extrapair partner present in neighborhood in year_*x* + 1_ or not	Blue tits may engage in EPCs with previous extrapair partners.	Individuals that have a previous extrapair partner in their close neighborhood might have more EPP.	

^a^∆ refers to the change between breeding seasons, calculated either as proportional change (year_*x* + 1_/year_*x*_) in the trait or as the difference (year_*x* + 1_ – year_*x*_) in the trait.

The quality of the social partner might be an important aspect influencing the decision of a focal individual to engage in extrapair mating. For instance, a weak pair bond resulting from behavioral incompatibility between the partners (Ihle et al. 2015) or genetic quality and/or compatibility (Foerster et al. 2003) could influence extrapair behavior. Furthermore, the tendency of an individual to engage in extrapair behavior might also influence the extrapair behavior of its partner (Maldonado-Chaparro et al. 2018). Thus, we also examined whether the occurrence of EPP is more consistent between years when the focal individual breeds with the same partner. Furthermore, past studies reported that divorced blue tits might still have extrapair young with their previous partner (Valcu and Kempenaers 2008; Gilsenan et al. 2017). Thus, for individuals paired with a different social partner, we assessed whether changes in levels of EPP depended on the presence of the former partner in the neighborhood.

Changes in EPP between years may also be explained by changes in the phenotypic composition of the breeding neighbors. For example, in blue tits, adult (compared to yearling) and larger males are more successful in siring extrapair young (Kempenaers et al. 1997; Schlicht et al. 2015a). Because most extrapair young are sired by first- or second-order neighbors (Schlicht et al. 2015a), the number or proportion of large, adult male neighbors may influence the likelihood that a pair has extrapair young in their nest or for a focal male to sire extrapair young in a neighboring nest (but see Roth et al. 2019). Similarly, there is competition among females (Kempenaers 1994; Midamegbe et al. 2011). A neighborhood containing a higher proportion of adult and larger females (i.e., potentially dominant or stronger females) may influence the likelihood that a focal female can obtain extrapair copulations with a neighboring male. Furthermore, individuals breeding in the same area over multiple years might be familiar with some of the neighbors from previous breeding seasons. Familiarity might influence the decision to engage in extrapair behavior or it might increase the chances to obtain extrapair copulations, for example, if it leads to reduced territorial conflicts and allows more extraterritorial visits, thereby facilitating meeting potential extrapair partners (Beletsky and Orians 1989; Grabowska-Zhang et al. 2011; Beck et al. 2020). Thus, we examine whether a higher proportion of familiar females and males and the presence of former extrapair partners influence changes in patterns of EPP.

## MATERIALS AND METHODS

### Study species and population

Blue tits are small, hole-nesting songbirds that breed only once per year (except for some replacement clutches) and that engage frequently in extrapair mating (about half of the broods contain at least one extrapair young and 10–15% of all offspring are sired by extrapair males; Kempenaers et al. 1992; Kempenaers et al. 1997; Delhey et al. 2003). Roughly half of the individuals breed in multiple years with the same social partner (Valcu and Kempenaers 2008; Gilsenan et al. 2017).

For this study, we use data on EPP from a population that breeds in a mixed-deciduous, oak-dominated forest close to Landsberg am Lech, Germany (“Westerholz,” 48°08’N 10°53′E, c. 40 ha; see also Schlicht et al. 2012). In 2007, we put up 277 wooden, small-holed (diameter 26 mm) nest-boxes at the site and studied the breeding behavior of the blue tits nesting in the boxes (60–176 pairs per year). Nest-boxes were distributed evenly across the site and placed approximately 40 m apart. Permits were obtained from the Bavarian government and the Bavarian regional office for forestry Landesanstalt für Wald und Forstwirtschaft (LWF).

### Assessment of EPP

We took blood samples (circa 10 µL) from all nestlings (at the age of 14 days) and breeding adults (which we caught inside the nest-box or with mist nets either during the breeding season or in the preceding winter) and we collected all unhatched eggs and dead nestlings for genotyping. Some unhatched eggs could not be genotyped and some nestlings disappeared from the nest and were not sampled (in 23% of nests at least one egg was not genotyped). We used 14 microsatellite markers and one sex chromosome-linked marker (ADCbm; ClkpolyQ; Mcµ4; PAT MP 2–43; Pca3, Pca4, Pca7, Pca8, and Pca9; Phtr3; PK11 and PK12; POCC1 and POCC6; and the sex chromosome-linked P2/P8). Microsatellite amplifications were performed in multiplexed PCRs (each 10-μL multiplex PCR contained 20–80 ng DNA) and primer mixes containing two to five primer pairs. Overall, we genotyped 10 227 out of 11 624 laid eggs (88%; between-year range: 80–97%) and compared the genotypes of parents and their offspring using the software CERVUS (Kalinowski et al. 2007). For each breeding season, we assigned to each male how many extrapair young he sired and, for each female, how many extrapair-sired eggs her clutch contained. For both sexes, we also determined the number of extrapair partners. Although the majority of the fertilized eggs were genotyped, the observed patterns of EPP may not be identical with the actual patterns.

### Measurements of changes in the local environment

For each focal individual (females and males separately), we examined the following changes in the local breeding environment over subsequent years (Table 1; Supplementary Figure S1).

#### Territory size

We estimated the size of the breeding territory (in square meters) using the r package “expp” (Valcu and Schlicht 2013; Schlicht et al. 2015a). The package assigns each point in the study area to the nearest breeding pair, thereby creating distinct territories using Thiessen polygons (Valcu and Kempenaers 2010; Schlicht et al. 2014; see Supplementary Figure S1). We then calculated changes in territory size by dividing the size in year *x* + 1 by the size in year *x* (ratio). We also calculated the difference in absolute territory size. We report the results using the proportional change in territory size. However, we repeated all analyses with the absolute change in territory size (see Supplementary Tables S1–S3).

#### Social partner

We examined whether or not the focal individual bred with a new partner in year *x* + 1 (binary variable: yes or no) and further assessed whether a former social partner was still breeding nearby in the first-order neighborhood (i.e., all neighbors whose territories adjoin the focal individuals’ territory borders) or not and tested whether this had an effect on the likelihood of having EPP. Furthermore, we calculated the change in body size of the social male by calculating the difference in tarsus length between the year *x* + 1 social male and the year *x* social male (analysis of female EPP; see Table 1).

#### The local neighborhood

We calculated the number of neighbors using the r package “expp” (see above). Based on the estimated territory distribution, we defined first-order (direct) neighbors as all territories sharing the focal pair’s territory border, and second-order neighbors as territories where one territory was in between. We calculated changes in the number of first-order neighbors by dividing the measure in year *x* + 1 by the measure in year *x* (see Table 1; Supplementary Figure S1). In the main text, we report the results of analyses using this ratio. However, we repeated all analyses using the absolute change in the number of first-order neighbors (Supplementary Table S1–S3). We also examined changes in the phenotypic composition of the neighborhood by calculating the average age and tarsus length of the direct neighbors (males or females). We assigned age as a binary variable (yearling = 1; adult = 2). The change was then calculated as the difference between year *x* + 1 and year *x*. Finally, we examined the change in the proportion of familiar female and male neighbors. We defined two birds as being familiar to each other when they had bred together (former partner after divorce), were previous extrapair partners, or had been first-order neighbors in previous years. For each focal individual, we then quantified for each year the proportion of familiar males and females in the local neighborhood and whether a former social or extrapair partner was present. We calculated changes as the difference in the proportion of familiar birds between year *x* + 1 and year *x*.

Investigating changes in EPP between years in relation to changes in the breeding environment might also shed light on the general but little-understood effect that older males are more successful in siring extrapair young (Cleasby and Nakagawa 2012; Schlicht et al. 2015a; Hsu et al. 2017). When yearlings turn adult, there might be specific changes in the environment causing this effect. For example, as yearlings, by definition, none of the neighbors are familiar and no previous breeding partner can be around. To investigate such age-specific changes, we ran two separate analyses: one for males that turned from yearling to adult and one including only adult males.

For all analyses, we only considered first-order neighbors because 1) individuals typically meet near territory borders, 2) most extraterritorial nest-box visits are with direct neighbors (Schlicht et al. 2015b), and 3) the probability that a female and a male have extrapair young together strongly decreases with increasing breeding distance (see Schlicht et al. 2015b; in our data set, 61% of the EP partners are first-order neighbors and 23% are second-order neighbors). Repeating the analyses with second-order neighbors included did not qualitatively change any of the conclusions (results not shown).

### Data selection and statistical analysis

For all statistical analyses, we used the software R 3.5.1 (R Core Team 2018).

#### Repeatability of EPP

We used data from all individuals that bred in our study area in at least 2 years and for which information on EPP was available (*N*_Males_ = 221, *N*_Females_ = 233). For males and females separately, we calculated the repeatability of 1) the number of extrapair partners, 2) the total number of extrapair young obtained by an individual, and 3) the occurrence of EPP (yes/no) within a given breeding season. For males, we additionally examined the repeatability in paternity loss, that is, in 4) the proportion of young in the male’s nest that were sired by another male (number of extrapair young/total number of young) and in 5) the occurrence of paternity loss (yes/no). We calculated repeatability for different measures of EPP because they have different biological meanings. For instance, high repeatability in the occurrence of EPP may indicate that some females and males are more likely to engage in extrapair behavior than others. The number of extrapair young sired by males refers directly to gains in reproductive success, whereas the number of extrapair young in a clutch represents both female behavior and her social mate’s reproductive loss. The number of extrapair young may be influenced by the relative number and timing of within-pair and extrapair copulations but also by postcopulatory mechanisms and, hence, may depend more on female identity than on male identity.

We fitted a generalized linear mixed-effect model (GLMM) using the rpt function of the R package “rptR” (Stoffel et al. 2017) with a Poisson distribution for the models using the dependent variables 1) and 2), proportion data for 4) and binary data for the models using variables 3) and 5). As random intercept, we included individual identity. We repeated the models, including additionally either the box identity or the pair identity as random intercept to control for variation explained by the location (nest-box) or the pair. We calculated the repeatability coefficient R, its 95% confidence interval (CI), and the associated *P*-value using 1000 bootstrapping runs. We report all repeatability estimates only on the original scale approximation as estimates did not differ considerably compared to the link-scale approximation (Nakagawa and Schielzeth 2010; Stoffel et al. 2017). For females, we repeated the analyses on a subset of individuals for which all eggs had been genotyped (*N*_Female_ = 83) to exclude a bias in the repeatability estimates due to incomplete sampling. Additionally, we calculated adjusted repeatabilities for females by including clutch size as a fixed effect and individual identity as random intercept. We included clutch size to control for the fact that extrapair young are usually found among the first-laid eggs (Magrath et al. 2009), and we would, thus, expect a lower proportion of extrapair young with increasing clutch size. Furthermore, clutch size gives an upper limit to the number of extrapair offspring. For males, we calculated adjusted repeatabilities by adding territory location (central or edge territory) as fixed effect, assuming that males breeding on the edge of the study area were more likely to have sired young in unsampled nests. As random intercept, we included individual identity. We also included male age as a fixed effect because adults are more likely to sire extrapair young than yearlings (Schlicht et al. 2015a).

#### Effects of changes in the breeding environment

To relate between-year changes in EPP to changes in the breeding environment, we only included individuals that were breeding in consecutive years and for which all relevant information of the breeding environment (Table 1) was available for both years (*N*_Males_ = 203, *N*_Females_ = 190). We tested our general hypothesis that between-year changes in the local breeding environment can explain changes in levels of EPP in females and males by examining the response variables 1) change in the number of extrapair partners, 2) change in the total number of extrapair young, and 3) change in status (i.e., individuals that had no extrapair young in year *x* but did so in year *x* + 1 or vice versa compared to individuals that did or did not have extrapair offspring in both years). We did not examine whether between-year changes in the local breeding environment can explain changes in paternity loss in males as paternity loss likely depends on the female perspective rather than on changes within the males’ local neighborhood.

For the variables “number of extrapair partners” and “number of extrapair young,” we calculated for each individual the difference between year *x* + 1 and year *x* and used this as the dependent variable in a linear mixed-effect model (LMM; package “lme4”; Bates et al. 2014). For females, we included 12 fixed effects describing changes in their breeding environment (see Table 1). We calculated correlation coefficients between all fixed effects to check for collinearity (Dormann et al. 2013). As none of the parameters strongly correlated (all *r* < 0.5; see Supplementary Tables S4 and S5), we included all into our models. As random effects, we included individual identity and year. For males, we constructed two models for each response variable: one including only individuals that turned from yearling to adult (*N* = 172) and one only including adult individuals (*N* = 49). We included nine fixed effects describing changes in the males’ breeding environment (see Table 1) and verified potential correlations as described above (all *r* < 0.5). As random effects, we included individual identity and year in the models for adult males, but only year in the model for “yearling to adult” because each individual only appeared once in that data set.

For the dependent variable “change in EPP status (yes/no),” we fitted GLMMs (package “lme4”; Bates et al., 2014) with a binomial error structure and a logit-link function. For both sexes, we included the same fixed effects as described for the previous models. However, in this case, we used absolute values because we examined whether a change in any of the environmental variables can explain a change in EPP status, regardless of the direction of that change (i.e., an increase or a decrease). All model results include adjusted approximations of the *P*-values based on multiple comparisons of predictors using the “glht” function of the “multcomp” package (Hothorn et al. 2008).

## RESULTS

### Repeatability of EPP

For females, the repeatability of the occurrence and the number of extrapair young in her clutch was small, but significant, and increased when only completely genotyped clutches were included (Table 2). Accounting for the effect of clutch size did not affect the results (Table 2). The number of extrapair sires was not significantly repeatable, even when only completely genotyped clutches were considered (Table 2).

**Table 2:** Repeatability of EPP (total number of extrapair young, number of extrapair mates, and the occurrence of EPP) for male and female blue tits and the repeatability of paternity loss in males (i.e., the proportion of young lost and the occurrence of paternity loss). Shown are the repeatability coefficients (*R*), their range, their 95% CIs and the associated *P*-values. *R*_adj_ refers to models controlling for the fixed effects territory location (central vs. edge), male age (yearling vs. adult), or clutch size. For females, results on the repeatability of EPP are once shown for all data and once only including completely genotyped clutches. Significant *P*-values are indicated in bold

	*R*	Range	95% CI	*P*	Fixed effect	*R*_adj_	Range	95% CI	*P*
Males									
Number of EPY	0.03	0.00–0.11	0.00–0.06	0.12	Location	0.03	0.00–0.10	0.00–0.05	0.12
					Age	0.06	0.00–0.21	0.00–0.13	**0.04**
Number of EP mates	0.07	0.00–0.24	0.00–0.14	0.08	Location	0.07	0.00–0.22	0.00–0.14	0.08
					Age	0.10	0.00–0.32	0.00–0.19	**0.03**
EPP occurrence	0.08	0.00–0.23	0.00–0.14	**0.02**	Location	0.08	0.00–0.20	0.00–0.14	**0.02**
					Age	0.10	0.00–0.23	0.00–0.15	**0.01**
Proportion of young lost	0.00	0.00–0.04	0.00–0.01	1.00	Location	0.00	0.00–0.03	0.00–0.01	1.00
					Age	0.00	0.00–0.23	0.00–0.01	1.00
Paternity loss	0.01	0.00–0.13	0.00–0.07	0.34	Location	0.01	0.00–0.14	0.00–0.08	0.34
					Age	0.02	0.00–0.23	0.00–0.08	0.31
Females									
Number of EPY									
All	0.12	0.00–0.35	0.00–0.19	**0.003**	Clutch size	0.10	0.00–0.26	0.00–0.18	**0.004**
Complete	0.33	0.00–0.84	0.06–0.61	**<0.001**	Clutch size	0.33	0.00–0.78	0.05–0.57	**<0.001**
Number of EP mates									
All	0.00	0.00–0.15	0.00–0.06	1.00	Clutch size	0.00	0.00–0.12	0.00–0.06	1.00
Complete	0.09	0.00–0.31	0.00–0.24	0.11	Clutch size	0.10	0.00–0.31	0.00–0.22	0.11
EPP occurrence									
All	0.10	0.00–0.22	0.00–0.15	**0.01**	Clutch size	0.09	0.00–0.19	0.00–0.14	**0.01**
Complete	0.25	0.00–0.55	0.03–0.38	**0.003**	Clutch size	0.22	0.00–0.75	0.02–0.39	**0.003**

For males, the between-year repeatability of the different measures of EPP was low (Table 2). The occurrence of EPP, that is, whether a male sired extrapair offspring or not, was significantly repeatable, while the number of extrapair young sired, the number of extrapair partners, and paternity loss were not (Table 2). Repeatability values did not change when controlling for territory location or age (Table 2).

Repeatability estimates were somewhat higher in females than in males, but the CIs overlapped for all metrics (Table 2). Hence, these differences may not be biologically meaningful. For both sexes, repeatability values for location (nest-box) and the specific partner (pair identity) were close to 0 (Supplementary Tables S6 and S7).

### Effects of changes in the breeding environment

**Figure 1:**
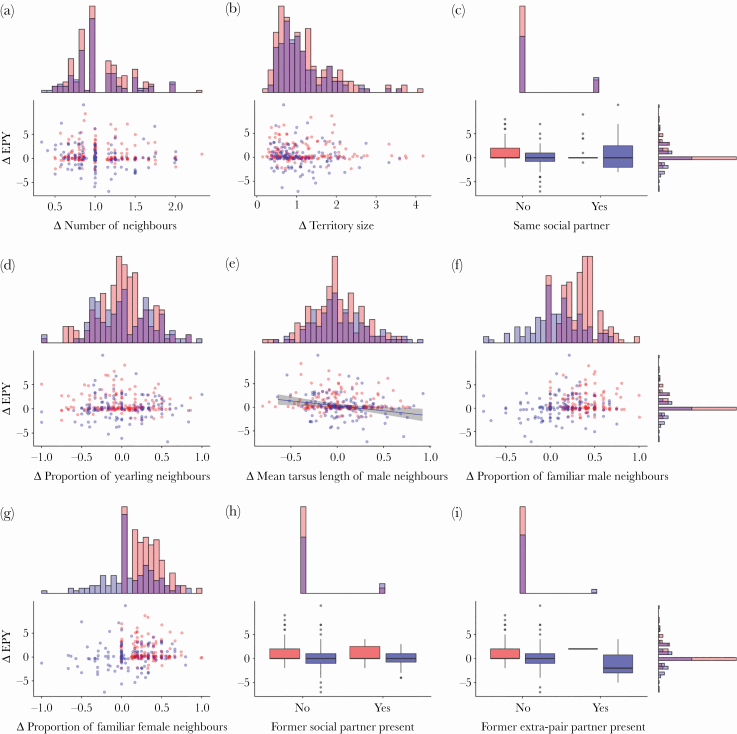
Between-year changes (Δ) in the number of extrapair young a male blue tit sired in relation to changes in the local breeding environment. (a) Change in the number of neighbors (range, yearling to adult = 0.50–2.30; mean, yearling to adult = 1.08; range, only adult = 0.33–2.00; mean only adult = 1.05); (b) change in territory size (range, yearling to adult = 0.15–4.18; mean, yearling to adult = 1.16; range, only adult = 0.34–3.59; mean, only adult = 1.08; estimated based on Dirichlet tiles); (c) change of social partner (yes/no); (d) change in the proportion of yearling male neighbors (range, yearling to adult = −1.00–0.86; mean, yearling to adult = −0.02; range, only adult = −1.00–1.00; mean, only adult = 0.01); (e) change in the mean tarsus length of male neighbors (range, yearling to adult = −0.80–0.76; mean, yearling to adult = 0.02; range, only adult = −0.64–0.93; mean, only adult = 0.06); (f) change in the proportion of familiar male neighbors (range, yearling to adult = 0.00–1.00; mean, yearling to adult = 0.35; range, only adult = −0.75–0.75; mean, only adult = 0.09); (g) change in the proportion of familiar female neighbors (range, yearling to adult = 0.00–1.00; mean, yearling to adult = 0.27; range, only adult = −1.00–0.80; mean, only adult = 0.07); (h) whether the former social partner was still present in the neighborhood (yes/no); (i) whether a former extrapair partner was still present in the neighborhood (yes/no). Individuals that turned from yearling to adult (*N* = 172) are shown in red, adult males (*N* = 49) are shown in blue. In (c), (h), and (i), boxplots show the minimum values, lower quartile, median, upper quartile, maximum values, and outliers ((c): yearling to adult: no = 150 cases, yes = 22; only adult: no = 98, yes = 26; (h): yearling to adult: no = 162, yes = 10; only adult: no = 106, yes = 18); (i): yearling to adult: no = 171, yes = 1; only adult: no = 116, yes = 8). We found a significant relationship between the mean tarsus length of male neighbors and changes in the number of EPY gained for adult males, which is why we added in (e) a linear regression line (in blue) and 95% CIs from the LMM described in the main text (in gray). See Methods for variable and model definitions and Tables 3 and 4 for results of statistical analyses.

For males, we found considerable variation in the between-year changes in the number of extrapair partners (from −4 to + 5; mean = 0.2 ± 1.0 standard deviation [SD]) and in the number of extrapair young sired (from −8 to + 11; mean = 0.5 ± 2.3 SD). However, these changes or the change in status were generally not predicted by changes in the local environment (Tables 3 and 4; Figure 1), neither for males that turned from yearling to adult nor for adult males that bred in multiple years. Only one effect was significant: a decrease in the average body size of male neighbors was associated with an increase in the total number of extrapair young sired (LMM estimate ± standard error [SE]: −2.10 ± 0.72, *P* = 0.03).

**Table 3:** Effects of changes in the local social environment on between-year changes in levels of EPP for yearling male blue tits that become adult (*N* = 172). EPP is measured as the change in the number of females with whom a male sired extrapair offspring (EP females), the number of young a male sired (EPY) and whether a male changed its’ EPP status (i.e., changed or remained the same). See Methods for details on the models

	∆ EP females			∆ EPY			Change in EPP status		
	Estimate ± SE	*t*	*P*	Estimate ± SE	*t*	*P*	Estimate ± SE	*t*	*P*
Intercept	0.56 ± 0.27			1.12 ± 0.58			−0.20 ± 0.72		
Number of neighbors	0.01 ± 0.20	0.04	1.00	−0.15 ± 0.44	−0.35	0.99	−0.18 ± 0.51	−0.35	0.99
Territory size	−0.18 ± 0.10	−1.89	0.37	−0.16 ± 0.21	−0.76	0.99	−0.43 ± 0.26	−1.67	0.54
Consistent social partner	−0.09 ± 0.10	−0.85	0.98	0.02 ± 0.22	0.10	1.00	−0.26 ± 0.26	−0.97	0.96
Proportion yearling male neighbors	−0.02 ± 0.22	−0.07	1.00	−0.43 ± 0.49	−0.88	0.97	0.44 ± 0.80	0.55	0.99
Average male neighbor tarsus length	−0.17 ± 0.24	−0.72	0.99	−0.53 ± 0.52	−1.01	0.94	−1.09 ± 0.97	−1.13	0.90
Proportion familiar males	0.40 ± 0.38	1.06	0.93	0.62 ± 0.81	0.77	0.99	1.26 ± 0.89	1.42	0.73
Proportion familiar females	−0.14 ± 0.37	−0.36	0.99	−0.25 ± 0.81	−0.31	0.99	−0.28 ± 0.90	−0.31	0.99
Previous social partner present	0.02 ± 0.30	0.07	1.00	0.003 ± 0.66	0.01	1.00	0.18 ± 0.71	0.25	0.99
Previous extrapair partner present	Not applicable as a previous extrapair partner was only present in one case								

**Table 4:** Effects of changes in the local environment on between-year changes in levels of EPP for adult male blue tits (*N* = 49). EPP is measured as the change in the number of females with whom a male sired extrapair offspring (EP females), the number of young a male sired (EPY), and whether a male changed its EPP status (i.e., changed or remained the same). See Methods for details on the models. Significant *P*-values are indicated in bold

	∆ EP females			∆ EPY			Change in EPP status		
	Estimate ± SE	*t*	*P*	Estimate ± SE	*t*	*P*	Estimate ± SE	*t*	*P*
Intercept	0.45 ± 0.27			1.41 ± 0.73			−0.03 ± 0.82		
Number of neighbors	−0.36 ± 0.23	−1.56	0.65	−1.07 ± 0.61	−1.74	0.52	−0.67 ± 0.59	−1.14	0.92
Territory size	−0.09 ± 0.08	−1.10	0.93	−0.14 ± 0.23	−0.61	0.99	0.37 ± 0.32	1.16	0.91
Consistent social partner	−0.01 ± 0.10	−0.13	1.00	0.25 ± 0.26	0.93	0.98	0.10 ± 0.25	0.40	0.99
Proportion yearling male neighbors	0.59 ± 0.25	2.31	0.17	0.98 ± 0.72	1.36	0.80	−0.77 ± 0.92	−0.84	0.99
Average male neighbor tarsus length	−0.58 ± 0.27	−2.15	0.24	−2.10 ± 0.72	−2.90	**0.03**	0.12 ± 0.99	0.12	1.00
Proportion familiar males	0.62 ± 0.36	1.73	0.52	1.32 ± 0.97	1.36	0.80	0.15 ± 1.06	0.14	1.00
Proportion familiar females	0.51 ± 0.30	1.71	0.54	0.67 ± 0.80	0.84	0.99	0.57 ± 1.02	0.55	0.99
Previous social partner present	−0.35 ± 0.24	−1.45	0.73	−0.87 ± 0.65	−1.33	0.82	0.30 ± 0.55	0.55	0.99
Previous extrapair partner present	−0.56 ± 0.34	−1.68	0.56	−1.62 ± 0.90	−1.80	0.47	−1.97 ± 1.15	−1.71	0.54

**Figure 2:**
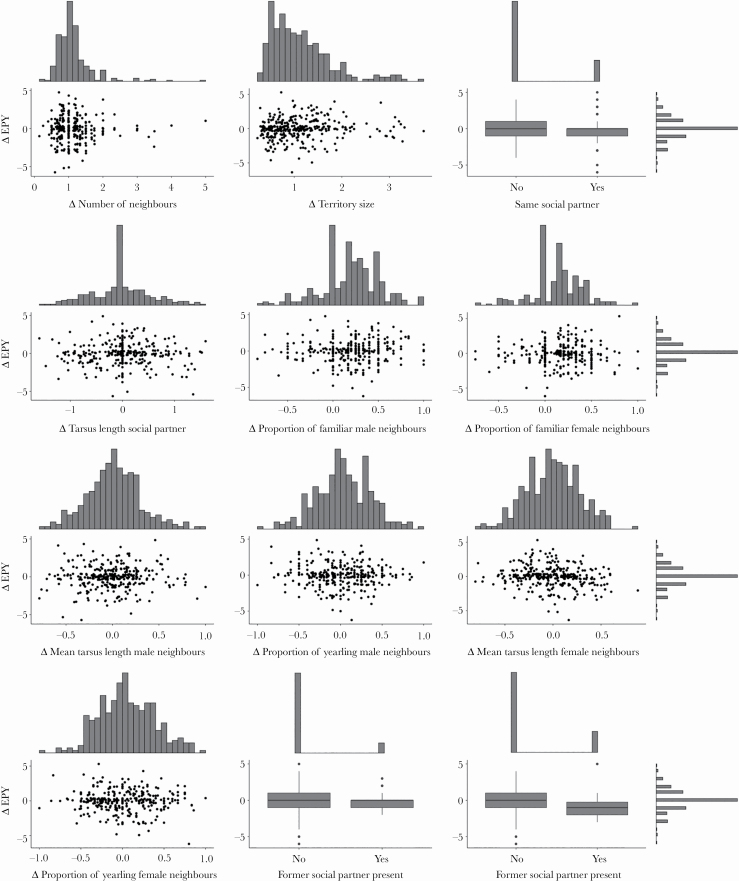
Between-year changes (Δ) in the number of extrapair young in a female blue tit’s clutch in relation to changes in the local breeding environment (*N* = 190 females). (a) Change in the number of neighbors (range = 0.14–5.00; mean = 1.13); (b) change in territory size (estimated based on Dirichlet tiles; range = 0.23–3.72; mean = 1.12); (c) change of social partner (yes/no); (d) change in tarsus length of the social partner (range = −1.59–1.60; mean = 0.03); (e) change in the proportion of familiar male neighbors (range = −0.83–1.00; mean = 0.20); (f) change in the proportion of familiar female neighbors (range = −0.75–1.00; mean = 0.16); (g) change in the mean tarsus length of male neighbors (range = −0.79–1.00; mean = − 0.002); (h) change in the proportion of yearling male neighbors (range = −1.00–1.00; mean = −0.002); (i) change in the mean tarsus length of female neighbors (range = −0.81–0.88; mean = −0.02); (j) change in the proportion of yearling female neighbors (range = −1.00–1.00; mean = −0.02); (k) whether the former social partner was still present in the neighborhood (yes/no); (l) whether a former extrapair partner was still present in the neighborhood (yes/no). In (c), (k), and (l) box plots show the minimum values, lower quartile, median, upper quartile, maximum values, and outliers ((c): no = 248 cases, yes = 65; (k): no = 278, yes = 35; (l): no = 295, yes = 18). See Methods for variable definitions and Table 5 and Supplementary Table S3 for the results of statistical analyses.

For females, between-year changes in the number of extrapair partners varied between −2 to + 3 (mean = −0.04 ± 0.8 SD) and changes in the number of extrapair young varied between −6 and +5 (mean = −0.04 ± 1.5 SD). We found no evidence that changes in the local environment between years explained changes in levels of EPP (Table 5; Figure 2).

**Table 5:** Effects of changes in the local environment on between-year changes in levels of EPP for female blue tits (*N* = 190). EPP is measured as the number of males that sired extrapair offspring in the female’s clutch (EP males), the number of extrapair young in the clutch (EPY), and whether a female changed its EPP status (i.e., changed or remained the same). See Methods for details on the models

	∆ EP males			∆ EPY			Change in EPP status		
	Estimate ± SE	*t*	*P*	Estimate ± SE	*t*	*P*	Estimate ± SE	*t*	*P*
Intercept	−0.08 ± 0.12			0.001 ± 0.23			−0.99 ± 0.56		
Number of neighbors	−0.004 ± 0.09	−0.05	1.00	−0.06 ± 0.17	−0.36	0.99	−0.40 ± 0.26	−1.55	0.77
Territory size	0.10 ± 0.05	2.06	0.36	0.13 ± 0.10	1.34	0.89	0.17 ± 0.21	0.79	0.99
Consistent social partner	0.20 ± 0.11	1.83	0.54	0.11 ± 0.22	0.50	0.99	0.87 ± 0.39	2.24	0.26
Social partner body size	0.14 ± 0.08	1.83	0.54	−0.03 ± 0.15	−0.20	1.00	0.22 ± 0.39	0.56	0.99
Proportion familiar males	0.002 ± 0.18	0.01	1.00	0.35 ± 0.35	0.99	0.99	0.57 ± 0.64	0.89	0.99
Proportion familiar females	0.06 ± 0.21	0.29	1.00	−0.18 ± 0.41	−0.43	1.00	0.61 ± 0.70	0.87	0.99
Average male neighbor body size	0.07 ± 0.15	0.49	0.99	0.24 ± 0.29	0.83	0.99	1.18 ± 0.72	1.65	0.70
Proportion yearling male neighbors	0.12 ± 0.17	0.70	0.99	0.05 ± 0.33	0.15	0.99	−0.49 ± 0.73	−0.68	0.99
Average female neighbor body size	−0.31 ± 0.16	−1.91	0.48	−0.71 ± 0.32	−2.25	0.25	0.28 ± 0.80	0.35	0.99
Proportion yearling female neighbors	0.01 ± 0.16	0.05	1.00	−0.21 ± 0.32	−0.64	0.99	0.26 ± 0.68	0.39	0.99
Previous social partner present	0.003 ± 0.15	0.02	1.00	−0.10 ± 0.29	−0.33	1.00	0.22 ± 0.45	0.48	0.99
Previous extrapair partner present	−0.53 ± 0.20	−2.74	0.07	−0.83 ± 0.38	−2.19	0.28	−0.10 ± 0.59	−0.16	1.00

## DISCUSSION

Many studies aimed to determine the underlying causes of the observed individual variation in the expression of EPP (Schlicht et al. 2015a; Baldassarre et al. 2016; Johnsen et al. 2017; Edwards et al. 2018). However, our understanding of this variation remains limited. We studied changes in levels of EPP for the same individuals that bred in different years. Using a long-term data set from a blue tit population, we investigated 1) to what extent EPP patterns are repeatable for an individual and 2) whether between-year changes in the local breeding environment can explain within-individual changes in the measures of EPP. Overall, our results show a relatively low but significant repeatability of EPP patterns and little effect of changes in the local environment. Although we cannot exclude that other, unmeasured individual or environmental changes play a role, variation in levels of EPP may also be due to chance events, at least to some extent.

### Repeatability of EPP

Extrapair matings have the potential to increase the intensity of sexual selection if males with specific phenotypic traits are more successful in acquiring extrapair matings (Møller and Birkhead 1994; Webster et al. 1995). Alternatively, EPP can have no impact on the strength of sexual selection if, for instance, all males have an equal likelihood to gain extrapair young (Schlicht and Kempenaers 2011), or EPP may even decrease the strength of sexual selection if extrapair sires are often males that failed to acquire a social mate (Lebigre et al. 2012).

Estimates of repeatability can be used to evaluate the consistency of a trait and to provide an upper limit for its heritability and, hence, for the potential for (sexual) selection. Several studies have estimated the repeatability of different behaviors ranging from exploratory behavior to mate preferences and foraging (average of 759 repeatability estimates across different behaviors and species: *R* = 0.37; Bell et al. 2009). Repeatability estimates of EPP vary considerably (see above), with some reports of high estimates such as in female tree swallows (*Tachycineta bicolor*) for the proportion of extrapair young in the brood (*R* = 0.83) and the number of extrapair sires (*R* = 0.73), suggesting that these behaviors may be heritable and can undergo selection (Whittingham et al. 2006). The repeatability of patterns of EPP in blue tits was generally low for both sexes but significant for the occurrence and the number of extrapair young in females and for the occurrence of EPP in males, despite considerable between-year changes in the local breeding context (see data distributions in Figures 1 and 2; Table 2). The repeatability of EPP did not increase when individuals retained the same social partner between years and we also found no evidence that EPP levels were location specific (no effect of nest-box identity).

The low repeatabilities reported in our study may partly be due to measurement errors caused by incomplete sampling because repeatability estimates of female EPP levels increased somewhat when only completely genotyped clutches were included (Table 2). Our results suggest that females are, to some extent, consistent in the likelihood to have extrapair offspring and in the number of extrapair young they produce. The number of extrapair sires was not repeatable and may rather depend on aspects of the breeding neighborhood (e.g., the number of mates available) and the timing of extrapair copulations or the phenotypes of the extrapair male(s), including variation in sperm quality and quantity. In some other species, repeatability estimates were moderate to high (see above), further suggesting that female EPP is an individual-specific trait. The underlying cause of the significant repeatability in female EPP and potential targets of selection could be, for instance, individual differences in the tendency to engage in extrapair copulations (Forstmeier 2007) or individual differences in the frequency of within-pair copulations. Studies on the heritability of female extrapair behavior are rare. In song sparrows (*Melospiza melodia*), the proportion of extrapair young in a clutch showed an estimated heritability of 0.12 (Reid et al. 2010). In zebra finches (*Taeniopygia guttata*), the responsiveness to extrapair courtships was also heritable (*h*^2^ = 0.11; Forstmeier et al. 2011). However, more research will be needed to show that female EPP or the underlying behavioral traits are heritable.

In males, consistency in EPP loss or gain can indicate that specific individual characteristics increase the probability to successfully engage in extrapair copulations or to successfully defend paternity, which, in turn, may result in sexual selection. This would for instance be the case if females prefer to copulate with males of a specific phenotype (Weatherhead and Boag 1995; Yezerinac and Weatherhead 1997; Whittingham and Dunn 2016). Male blue tits showed significant repeatability only in whether they obtained extrapair offspring but not in the number of extrapair partners or in the number of extrapair young gained. This indicates that certain male phenotypes may consistently be more likely to sire extrapair young, while the number of offspring sired and with how many extrapair partners may depend more on the composition of the breeding environment (e.g., the availability of mates) or on postcopulatory mechanisms (i.e., sperm competition). Our findings and previous studies reported low repeatabilities for EPP in males (see Introduction), suggesting that EPP is not simply an individual-specific trait. Repeatability estimates in males might also be lower due to incomplete sampling. This is hard to avoid in natural populations because males may have sired extrapair young in broods that were not or not completely genotyped (e.g., abandoned clutches, broods in natural cavities in or outside the study area). To reduce this effect, we repeated the analyses controlling for territory location (i.e., edge or central territory), assuming that males breeding on the edge of the study site are more likely to sire young in unsampled broods. However, this did not change the repeatability estimates qualitatively (Table 2).

Taken together, the observed low repeatabilities of measures of EPP in both sexes suggest that EPP may not cause strong sexual selection. A previous study on blue tits showed that the contribution of EPP to variance in overall male reproductive success was small but significant (Schlicht and Kempenaers 2013). As expected, estimates of the potential for sexual selection were higher for males than for females, but opportunities for sexual selection may still be limited. The authors concluded that variation in reproductive success may largely be caused by stochastic processes and was unrelated to phenotypic traits, which is in line with our findings.

Studies on zebra finches in aviaries showed that the number of extrapair courtships (i.e., mating effort) performed by males and the responsiveness of females to extrapair courtships are highly repeatable, heritable traits that contribute to the occurrence of extrapair copulations and the resulting levels of EPP (Forstmeier 2004; Forstmeier 2007; Forstmeier et al. 2011). Thus, an alternative or additional explanation for the low repeatability reported in our study is related to the fact that most studies—including ours—only measure the outcome of extrapair behavior in terms of paternity. In natural systems, we still do not know to which extent variation in EPP patterns reflects variation in extrapair behavior of individuals and in the number of extrapair copulations they obtained. Many extrapair copulations may not lead to fertilizations (Hunter et al. 1992) and, hence, remain undetected (Girndt et al. 2018). EPP emerges from a series of behavioral and physiological processes involving multiple individuals. Thus, for an extrapair copulation to successfully fertilize an egg, other factors, such as the number and timing of within-pair copulations, ejaculate size, and the relative competitiveness of sperm from different males, will also play a role. These factors are hard if not impossible to control for but likely influence the observed levels of paternity and contribute to the “unexplained variation.” In most natural situations, accurately recording extrapair (and within-pair) copulations is not feasible (but see Hunter et al. 1992). However, individual repeatability in extrapair behavior can be investigated either in colony breeders (e.g., Hunter et al. 1992) or in a captive environment (e.g., Forstmeier 2004).

### Effects of changes in the local breeding environment

Most studies investigating the effects of the local environment on EPP considered among-individual variation within a given breeding season instead of within-individual variation across seasons. Such an approach does not allow to disentangle whether variation in EPP is caused by environmental or individual-specific differences. Here, we find that changes in the breeding environment between years had little effect on individual-level changes in the occurrence or frequency of EPP. We considered the effect of two potentially important aspects of the social context in which extrapair behavior occurs. First, we investigated the characteristics of the local neighborhood, that is, the phenotypic composition, in terms of male traits known to explain EPP patterns in blue tits within a given season (age and body size; Schlicht et al. 2015b), in terms of female traits potentially reflecting dominance or competitive ability (age and body size), and in terms of the familiarity of the focal individuals with their neighbors (proportion of familiar neighbors).

For adult males, a decrease in the average body size of their male neighbors was associated with a higher number of extrapair offspring sired (Table 4). In blue tits, extrapair males are typically larger than within-pair males (Kempenaers et al. 1997) and, hence, smaller males in the neighborhood might have increased the chances for a male to sire extrapair offspring. If this result is robust, it suggests that extrapair mating success may depend on the competitiveness of a male relative to its neighbors (male–male competition). In females, variation in the competitiveness of the breeding neighborhood did not explain between-year changes in EPP (Table 5). Similarly, in great tits, the phenotypic composition of the neighborhood (in this case, age and exploration behavior of both sexes) was not related to patterns of EPP within years (Roth et al. 2019). Although familiarity among neighbors could potentially also enhance the probability of extrapair copulations, we found no evidence for such effects.

Second, we investigated whether between-year changes in EPP could be explained by the presence of the social partner from the previous breeding season. We considered the effect of having the same or a different social partner or of having the former social partner still present in the local neighborhood. Neither of these factors explained changes in patterns of EPP in males or in females. Similarly, a study on patterns of EPP in two other blue tit populations in France (Charmantier and Blondel 2003) reported no effect of mate fidelity (i.e., breeding with the same or a different social partner). Furthermore, if mate fidelity plays a role, we would expect a higher repeatability of EPP for pairs as reported in coal tits (*Parus ater*); repeatability in the number of extrapair young produced was high for pairs staying together but decreased in cases of mate change (Dietrich et al. 2004). In our blue tit population, however, repeatability did not increase when social pairs were considered instead of individuals.

Other unmeasured individual and/or environmental aspects might explain variation in EPP. For instance, extrapair siring success in male blue tits has been related to plumage coloration or song characteristics (Delhey et al. 2006; Poesel et al. 2006, 2011). Thus, considering changes in the expression of these traits within the close neighborhood may better explain changes in EPP. Furthermore, these individual traits can change over the course of a lifetime. For instance, American redstarts (*Setophaga ruticilla*) were most colorful in their second breeding season (Marini et al. 2015; Reudink et al. 2015) and, in blue tits, crown coloration (Delhey and Kempenaers 2006) and song characteristics differ with age (Poesel et al. 2006). Investigating within-individual changes in such traits may potentially explain changes in extrapair success and could also shed light on the little-understood effect of male age on EPP. Finally, environmental factors, such as weather conditions (Bouwman and Komdeur 2006; Grant and Grant 2019) or food availability (Václav et al. 2003), may cause changes in the social structure (prior or during breeding) or in the costs of engaging in extrapair copulations and, consequently, may alter patterns of EPP.

EPP is inherently a social process involving several individuals. Thus, the probability to engage in extrapair copulations may be predicted better by recent interactions between individuals (i.e., between social pairs and potential extrapair partners) rather than by events from the previous breeding season or by individual-specific phenotypic traits. For instance, blue tits frequently interact in larger flocks during winter. These associations seem to play an important role in the formation of social pairs (Smith 1984; Culina 2014; Gilsenan et al. 2017), in extrapair associations (Beck et al. 2020), and in the composition of breeding neighborhoods (Firth and Sheldon 2016). Furthermore, it might be interesting to study the number and timing of interactions between close neighbors after settlement at the breeding box (i.e., when nest building has started) and during the fertile period of the female (Schlicht et al. 2015b). Such data would allow examining the intensity of mate guarding and effects of local breeding synchrony (i.e., the overlap in fertile period of females in the close neighborhood) in relation to patterns of EPP.

Lastly, we examined whether adult males sired more extrapair offspring than yearlings because they experienced a different (social) environment. Yearling males by definition breed for the first time, implying that they have no familiar neighbors from previous breeding seasons and no former partner(s) that can still breed nearby. However, we found no evidence for an effect of changes in the number of familiar neighbors from previous breeding seasons or in other aspects of the local environment on extrapair success either for males that bred first as yearling and then as adult or for adult males that bred in multiple years (Tables 3 and 4). A recent study on captive house sparrows showed that, although older males outperformed yearling males in siring extrapair offspring, yearling and adult males did not differ in their success in obtaining extrapair copulations (Girndt et al. 2018). However, adult males delivered almost three times more sperm to the female’s egg than young males (as estimated by counting sperm on the perivitelline membrane; Girndt et al. 2019), suggesting that postcopulatory mechanisms (sperm competition) may play a role rather than differences in local environment or male attractiveness.

## CONCLUSIONS AND FUTURE DIRECTIONS

This study shows that EPP in blue tits is somewhat repeatable, perhaps more so for females than for males. Individual-level changes in patterns of EPP between years were largely independent of changes in the local, social neighborhood, including changes in territory size (local breeding density), the identity of the social partner, and the composition of the neighborhood. Males, however, were more likely to sire extrapair young when their neighbors were smaller, an effect that—if true—suggests that the relative competitive ability of males is important. Alternatively, changes in other, unmeasured aspects of the local environment, such as associations or interactions between individuals prior to breeding, and individual qualities, such as plumage color or song characteristics, may be important determinants of EPP. Although the readiness to engage in extrapair behavior may be an individual-specific trait, EPP is the ultimate outcome of behavioral events and physiological processes involving several individuals. Therefore, variation in EPP may also depend, to some extent, on coincidental opportunities, such as “chance meetings” between two individuals that are willing to copulate and can do so without disturbance or other “chance events,” such as the exact timing of within-pair and extrapair copulations and the amount of sperm transferred.

## Supplementary Material

araa069_suppl_Supplementary_Material

## Notes

Data availability: Analyses reported in this article can be reproduced using the data provided by Beck, Valcu, et al. (2020).
