# Supplementary material for: Analysis of within-individual variation in extrapair paternity in blue tits (Cyanistes caeruleus) shows low repeatability and little effect of changes in neighborhood
Source: Behav Ecol. 2020 Oct 1;31(6):1303–15. doi: 10.1093/beheco/araa069 (PMC7689542; doi:10.1093/beheco/araa069)
Supplement: araa069_suppl_Supplementary_Material [file araa069_suppl_supplementary_material.docx]

**Supplementary material for:**

Analysis of within-individual variation in extra-pair paternity in blue tits (*Cyanistes caeruleus*) shows low repeatability and little effect of changes in neighborhood

Kristina B. Beck^1^, Mihai Valcu^1^, Bart Kempenaers^1*^

^1^ Department of Behavioural Ecology and Evolutionary Genetics, Max Planck Institute for Ornithology, Seewiesen, Germany


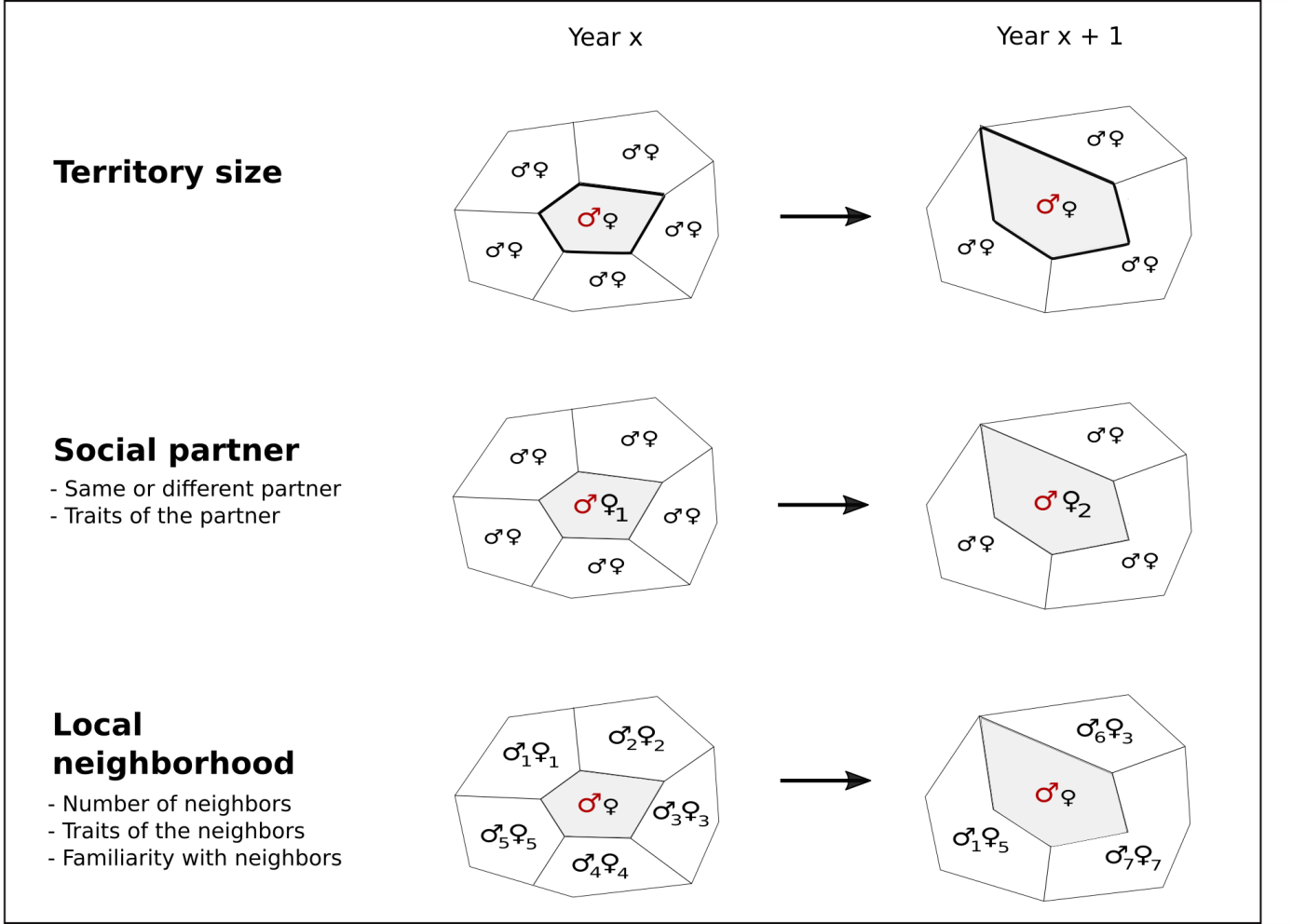


**Figure S1.** Schematic illustration of the different environmental contexts in which extra-pair paternity occurs that can change for an individual from one breeding season to the next (year x to year x+1). The focal individual (here a male) is represented in red, neighbors and the social partner in black. Breeding territories are represented by polygons. The corresponding context which might change between years is marked in bold. See also Table 1.

**Table S1.** Effects of changes in the local environment on between-year changes in levels of extra-pair paternity for yearling male blue tits that become adult (N=172). Extra-pair paternity is measured as the change in the number of females with whom a male sired extra-pair offspring (EP females), the number of young a male sired (EPY) and whether a male changed its’ EPP status (i.e., changed or remained the same). The change in territory size and the number of neighbors was here calculated as difference and not as proportion and both variables were standardized. See methods for details on the models.

|  | ∆ **EP females** | | |  | ∆ **EPY** | | |  | **Change in EPP status** | | |
| --- | --- | --- | --- | --- | --- | --- | --- | --- | --- | --- | --- |
|  | **Estimate ± SE** | **t** | **p** |  | **Estimate ± SE** | **t** | **p** |  | **Estimate ± SE** | **t** | **p** |
| Intercept | 0.36  ± 0.15 |  |  |  | 0.78  ± 0.32 |  |  |  | - 0.67  ± 0.55 |  |  |
| Number of neighbours | 0.01  ± 0.07 | 0.13 | 1.00 |  | -0.03  ±0.15 | -0.16 | 0.99 |  | -0.38  ±0.29 | -1.31 | 0.81 |
| Territory size | -0.13  ± 0.07 | -1.84 | 0.41 |  | -0.07  ±0.16 | -0.46 | 0.99 |  | 0.12  ±0.26 | 0.45 | 0.99 |
| Consistent social partner | -0.09  ± 0.10 | -0.83 | 0.98 |  | 0.03  ±0.22 | 0.14 | 1.00 |  | -0.23  ±0.26 | -0.86 | 0.98 |
| Proportion yearling male neighbours | -0.004  ± 0.22 | -0.02 | 1.00 |  | -0.42  ±0.49 | -0.87 | 0.98 |  | 0.30  ±0.79 | 0.39 | 0.99 |
| Average male neighbour tarsus length | -0.20  ± 0.24 | -0.82 | 0.98 |  | -0.53  ±0.52 | -1.02 | 0.94 |  | -1.07  ±0.98 | -1.09 | 0.92 |
| Proportion familiar males | 0.40  ± 0.38 | 1.07 | 0.92 |  | 0.60  ±0.81 | 0.73 | 0.99 |  | 0.96  ±0.87 | 1.10 | 0.91 |
| Proportion familiar females | -0.12  ±0.37 | -0.32 | 0.99 |  | -0.25  ±0.81 | -0.31 | 0.99 |  | -0.18  ±0.91 | -0.20 | 0.99 |
| Previous social partner present | -0.02  ± 0.31 | -0.07 | 1.00 |  | -0.02  ±0.66 | -0.03 | 1.00 |  | 0.27  ±0.71 | 0.38 | 0.99 |

**Table S2.** Effects of changes in the local environment on between-year changes in levels of extra-pair paternity for adult male blue tits (N=49). Extra-pair paternity is measured as the change in the number of females with whom a male sired extra-pair offspring (EP females), the number of young a male sired (EPY) and whether a male changed its’ EPP status (i.e., changed or remained the same). The change in territory size and the number of neighbors was here calculated as difference and not as proportion and both variables were standardized. See methods for details on the models.

|  | ∆ **EP females** | | |  | ∆ **EPY** | | |  | **Change in EPP status** | | |
| --- | --- | --- | --- | --- | --- | --- | --- | --- | --- | --- | --- |
|  | **Estimate ± SE** | **t** | **p** |  | **Estimate ± SE** | **t** | **p** |  | **Estimate ± SE** | **t** | **p** |
| Intercept | 0.07  ± 0.11 |  |  |  | 0.27  ± 0.32 |  |  |  | - 0.71  ± 0.58 |  |  |
| Number of neighbours | -0.13  ± 0.08 | -1.54 | 0.68 |  | -0.48  ±0.23 | -2.13 | 0.25 |  | 0.33  ±0.35 | 0.94 | 0.97 |
| Territory size | -0.10  ± 0.09 | -1.20 | 0.89 |  | -0.13  ±0.23 | -0.57 | 0.99 |  | 0.17  ±0.32 | 0.53 | 0.99 |
| Consistent social partner | -0.04  ± 0.10 | -0.36 | 0.99 |  | 0.19  ±0.26 | 0.71 | 0.99 |  | 0.11  ±0.25 | 0.45 | 0.99 |
| Proportion yearling male neighbours | 0.48  ± 0.26 | 1.87 | 0.41 |  | 0.74  ±0.72 | 1.03 | 0.95 |  | -0.96  ±1.00 | -0.96 | 0.97 |
| Average male neighbour tarsus length | -0.49  ± 0.27 | -1.81 | 0.46 |  | -1.86  ±0.72 | -2.58 | 0.08 |  | 0.53  ±1.00 | 0.53 | 0.99 |
| Proportion familiar males | 0.50  ± 0.36 | 1.41 | 0.77 |  | 1.05  ±0.96 | 1.09 | 0.93 |  | 0.60  ±1.15 | 0.52 | 0.99 |
| Proportion familiar females | 0.55  ± 0.30 | 1.82 | 0.46 |  | 0.71  ±0.80 | 0.88 | 0.98 |  | -0.08  ±1.08 | -0.08 | 1.00 |
| Previous social partner present | -0.34  ± 0.25 | -1.37 | 0.80 |  | -0.81  ±0.66 | -1.23 | 0.87 |  | 0.43  ±0.58 | 0.73 | 0.99 |
| Previous extra-pair partner present | -0.55  ± 0.34 | -1.64 | 0.59 |  | -1.56  ±0.90 | -1.73 | 0.53 |  | -1.82  ±1.17 | -1.56 | 0.66 |

**Table S3.** Effects of changes in the local environment on between-year changes in levels of extra-pair paternity for female blue tits (N=190). Extra-pair paternity is measured as the change in the number of males with whom a female sired extra-pair offspring (EP males), the number of young within the females clutch (EPY) and whether a female changed its’ EPP status (i.e., changed or remained the same). The change in territory size and the number of neighbors was here calculated as difference and not as proportion and both variables were standardized. See methods for details on the models.

|  | ∆ **EP males** | | |  | ∆ **EPY** | | |  | **Change in EPP status** | | |
| --- | --- | --- | --- | --- | --- | --- | --- | --- | --- | --- | --- |
|  | **Estimate ± SE** | **t** | **P** |  | **Estimate ± SE** | **t** | **P** |  | **Estimate ± SE** | **t** | **P** |
| Intercept | - 0.08  ± 0.06 |  |  |  | - 0.06  ± 0.13 |  |  |  | - 1.42  ± 0.50 |  |  |
| Number of neighbours | 0.02  ±0.03 | 0.69 | 0.99 |  | 0.003  ±0.05 | 0.06 | 1.00 |  | -0.09  ± 0.12 | -0.77 | 0.99 |
| Territory size | 0.08  ±0.05 | 1.60 | 0.72 |  | 0.08  ±0.10 | 0.86 | 0.99 |  | 0.23  ± 0.23 | 1.03 | 0.98 |
| Consistent social partner | 0.20  ±0.11 | 1.79 | 0.57 |  | 0.10  ±0.22 | 0.44 | 0.99 |  | 0.84  ± 0.39 | 2.17 | 0.30 |
| Social partner body size | 0.14  ±0.08 | 1.85 | 0.52 |  | -0.03  ±0.15 | -0.17 | 1.00 |  | 0.25  ± 0.39 | 0.64 | 0.99 |
| Proportion familiar males | -0.001  ±0.18 | -0.003 | 1.00 |  | 0.35  ±0.36 | 0.99 | 0.99 |  | 0.68  ± 0.64 | 1.07 | 0.98 |
| Proportion familiar females | 0.06  ±0.21 | 0.30 | 0.99 |  | -0.18  ±0.41 | -0.44 | 0.99 |  | 0.66  ± 0.70 | 0.95 | 0.99 |
| Average male neighbour body size | 0.09  ±0.15 | 0.61 | 0.99 |  | 0.26  ±0.29 | 0.88 | 0.99 |  | 1.09  ± 0.72 | 1.52 | 0.79 |
| Proportion yearling male neighbours | 0.10  ±0.17 | 0.60 | 0.99 |  | 0.03  ±0.33 | 0.10 | 1.00 |  | -0.50  ± 0.72 | -0.70 | 0.99 |
| Average female neighbor body size | -0.32  ± 0.16 | -1.98 | 0.42 |  | -0.73  ± 0.32 | -2.30 | 0.22 |  | 0.34  ± 0.80 | 0.42 | 0.99 |
| Proportion yearling female neighbors | -0.001  ± 0.17 | -0.01 | 1.00 |  | -0.23  ± 0.32 | -0.72 | 0.99 |  | 0.30  ± 0.67 | 0.45 | 0.99 |
| Previous social partner present | 0.004  ±0.15 | 0.03 | 1.00 |  | -0.09  ±0.29 | -0.32 | 0.99 |  | 0.17  ± 0.45 | 0.38 | 0.99 |
| Previous extra-pair partner present | -0.55  ±0.20 | -2.80 | 0.06 |  | -0.85  ±0.38 | -2.22 | 0.26 |  | -0.09  ± 0.59 | -0.15 | 1.00 |

**Table S4.** Table showing the correlation coefficients of all fixed effects included in the female model (excluding the categorical variables). In addition, we calculated the variance inflation factor (VIF) for all fixed effects in each model (including the categorical variables). The VIF was well below the threshold in all cases (ranging from 1.04 – 1.80; Dormann et al. 2013).

|  | Number of neighbors | Social partner body size | Territory size | Proportion familiar males | Proportion familiar females | Average male neighbor tarsus length | Proportion yearling male neighbors | Proportion yearling female neighbors |
| --- | --- | --- | --- | --- | --- | --- | --- | --- |
| Social partner body size | -0.010 |  |  |  |  |  |  |  |
| Territory size | 0.297 | 0.074 |  |  |  |  |  |  |
| Proportion familiar males | -0.046 | 0.056 | 0.020 |  |  |  |  |  |
| Proportion familiar females | -0.089 | -0.046 | -0.003 | 0.418 |  |  |  |  |
| Proportion yearling male neighbors | 0.038 | -0.018 | -0.155 | -0.419 | -0.104 |  |  |  |
| Average male neighbor tarsus length | -0.041 | -0.072 | -0.066 | -0.057 | -0.007 | -0.016 |  |  |
| Proportion yearling female neighbors | 0.008 | -0.021 | -0.272 | -0.055 | -0.340 | 0.101 | 0.405 |  |
| Average female neighbor body size | 0.018 | 0.090 | -0.105 | -0.039 | -0.154 | 0.090 | 0.070 | 0.098 |

**Table S5**. Table showing the correlation coefficients of all fixed effects included in the models on yearling males turning adult and only adults (in italic), excluding the categorical variables. In addition, we calculated the variance inflation factor (VIF) for all fixed effects for each model (including the categorical variables). The VIF was well below the threshold in all cases (Range in males turning from yearling to adult: 1.02 – 1.39; only adults: 1.04 – 1.79; Dormann et al. 2013).

|  | Number of neighbors | Territory size | Proportion familiar males | Proportion familiar females | Proportion yearling male neighbors |
| --- | --- | --- | --- | --- | --- |
| Territory size | 0.159  *0.154* |  |  |  |  |
| Proportion familiar males | -0.052  *-0.135* | 0.172  *-0.025* |  |  |  |
| Proportion familiar females | -0.034  *-0.151* | 0.071  *0.109* | 0.431  *0.439* |  |  |
| Proportion yearling male neighbors | 0.123  *0.054* | -0.125  *-0.129* | -0.341  *-0.409* | -0.158  *-0.131* |  |
| Average male neighbor tarsus length | -0.112  *-0.139* | -0.001  *-0.074* | 0.077  *-0.101* | 0.057  *-0.073* | -0.059  *0.006* |

**Table S6**. Repeatability of extra-pair paternity (total number of extra-pair young, number of extra-pair mates and the occurrence of extra-pair paternity) for male and female blue tits and the repeatability of paternity loss in males (i.e., the number of young lost and the occurrence of paternity loss). Shown are the repeatability coefficients (R), their 95% confidence intervals (CI) and the associated P-values for the two random intercepts „individual identity“ and „box identity“.

|  | **Individual** | |  |  | **Box** |  |  |
| --- | --- | --- | --- | --- | --- | --- | --- |
|  | **R** | **95%CI** | **P** |  | **R** | **95%CI** | **P** |
| **Males** |  |  |  |  |  |  |  |
| Number of EPY | 0.03 | 0.00 - 0.06 | 0.12 |  | 0.00 | 0.00 – 0.02 | 1.00 |
|  |  |  |  |  |  |  |  |
| Number of EP mates | 0.06 | 0.00 - 0.15 | 0.14 |  | 0.03 | 0.00 – 0.10 | 0.27 |
| EPP occurrence | 0.07 | 0.00 – 0.13 | 0.07 |  | 0.02 | 0.00 – 0.09 | 0.31 |
| Proportion of young lost | 0.00 | 0.00 – 0.02 | 0.50 |  | 0.01 | 0.00 – 0.03 | 0.10 |
| Paternity loss | 0.00 | 0.00 – 0.06 | 1.00 |  | 0.04 | 0.00 – 0.10 | 0.13 |
| **Females** | |  |  |  |  |  |  |
| Number of EPY | 0.12 | 0 - 0.21 | **0.003** |  | 0.04 | 0.00 - 0.11 | 0.12 |
| Number of EP mates | 0.00 | 0 - 0.05 | 0.50 |  | 0.00 | 0.00 - 0.05 | 1.00 |
|  |  |  |  |  |  |  |  |
| EPP occurrence | 0.10 | 0 – 0.15 | **0.01** |  | 0.00 | 0.00 – 0.04 | 1.00 |

**Table S7**. Repeatability of extra-pair paternity (total number of extra-pair young, number of extra-pair mates, the occurrence of extra-pair paternity) for male and female blue tits and the repeatability of paternity loss in males (i.e., the number of young lost and the occurrence of paternity loss). Shown are the repeatability coefficients (R), their 95% confidence intervals (CI) and the associated P-values for the two random intercept „individual identity“ and „pair identity“.

|  | **Individual** | |  |  | **Pair** |  |  |
| --- | --- | --- | --- | --- | --- | --- | --- |
|  | **R** | **95%CI** | **P** |  | **R** | **95%CI** | **P** |
| **Males** |  |  |  |  |  |  |  |
| Number of EPY | 0.01 | 0.00 - 0.02 | 0.40 |  | 0.02 | 0.00 – 0.20 | 0.34 |
|  |  |  |  |  |  |  |  |
| Number of EP mates | 0.04 | 0.00 - 0.11 | 0.22 |  | 0.09 | 0.00 – 0.32 | 0.18 |
| EPP occurrence | 0.06 | 0.00 - 0.11 | 0.09 |  | 0.00 | 0.00 – 0.04 | 1.00 |
| Proportion of young lost | 0.01 | 0.00 - 0.08 | 0.34 |  | 0.00 | 0.00 – 0.04 | 1.00 |
| Paternity loss | 0.00 | 0.00 - 0.05 | 1.00 |  | 0.00 | 0.00 – 0.21 | 0.50 |
| **Females** | |  |  |  |  |  |  |
| Number of EPY | 0.12 | 0.00 - 0.20 | **0.003** |  | 0.04 | 0.00 - 0.11 | 0.12 |
| Number of EP mates | 0.00 | 0.00 - 0.06 | 0.50 |  | 0.00 | 0.00 - 0.12 | 1.00 |
| EPP occurrence | 0.10 | 0.00 – 0.15 | **0.01** |  | 0.00 | 0.00 – 0.03 | 1.00 |

**References**

Dormann CF, Elith J, Bacher S, Buchmann C, Carl G, Carré G, Marquéz JRG, Gruber B, Lafourcade B, Leitão PJ. 2013. Collinearity: a review of methods to deal with it and a simulation study evaluating their performance. Ecography (Cop). 36:27–46.
